# A Case of Laryngeal Tuberculosis Mimicking Lymphoma

**DOI:** 10.7759/cureus.13744

**Published:** 2021-03-06

**Authors:** Henriette De La Garza, Rene Flores, Horiana B Grosu

**Affiliations:** 1 Dermatology, Boston University School of Medicine, Boston, USA; 2 Pulmonary Medicine, MD Anderson Cancer Center, Houston, USA; 3 Surgery, Beth Israel Deaconess Medical Center, Harvard Medical School, Boston, USA

**Keywords:** tuberculosis, laryngeal tuberculosis, lymphoma, laryngeal carcinoma

## Abstract

The incidence of laryngeal tuberculosis has steadily increased due to rising prevalence of HIV infection, immunosuppressive diseases and treatments, and the emergence of multidrug-resistant organisms and atypical mycobacteria. We report on a woman with a unique presentation of laryngeal tuberculosis mimicking lymphoma to remind clinicians that the diagnosis of laryngeal tuberculosis merits awareness and that delay in diagnosis poses a serious threat to the patient due to delayed treatment and further complications.

## Introduction

Laryngeal tuberculosis (TB) is rare, and requires a high degree of clinical suspicion to make the diagnosis. However, the incidence of laryngeal TB has steadily increased due to rising prevalence of HIV infection, immunosuppressive diseases and treatments, and the emergence of resistant organisms and atypical mycobacteria [1]. Delay in diagnosis of primary laryngeal TB poses a serious threat to the patient due to delayed treatment and further complications.

## Case presentation

**Figure 1:**
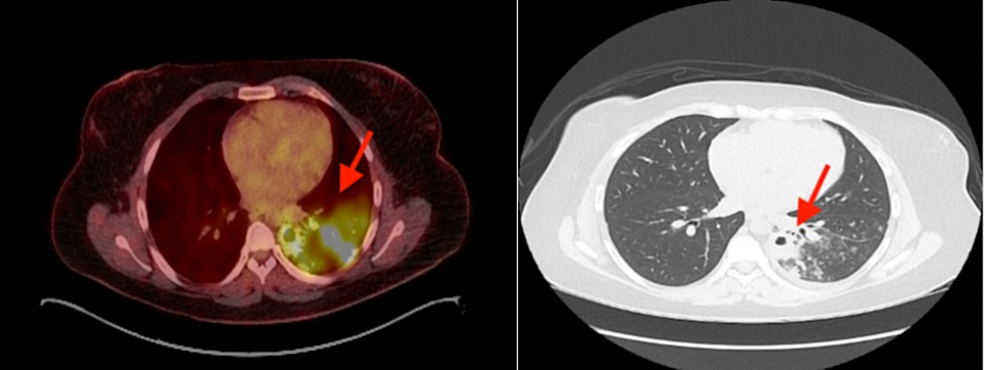
Chest positron emission tomography–computed tomography (PET-CT) showing significant pulmonary infiltrate with cavitary lesions.

A 31-year-old female from Guatemala presented with two months of worsening hoarseness and sore throat with no associated symptoms of fever, night sweats, weight loss, or dysphagia. Her past medical history was significant for a renal transplant in 2008 and she is currently on double immunosuppressive therapy. At time of presentation as part of the workup she underwent laryngoscopic examination that showed bilateral infiltrative process of bilateral true vocal cords. Biopsy of right and left vocal cord at an outside hospital reported nasal natural killer (NK)/T cell lymphoma. As a result, she presented to our institution for further evaluation. A positron emission tomography-computed tomography (PET-CT) of the chest was done and revealed significant pulmonary infiltrate with cavitary lesions. (Figure 1).

**Figure 2:**
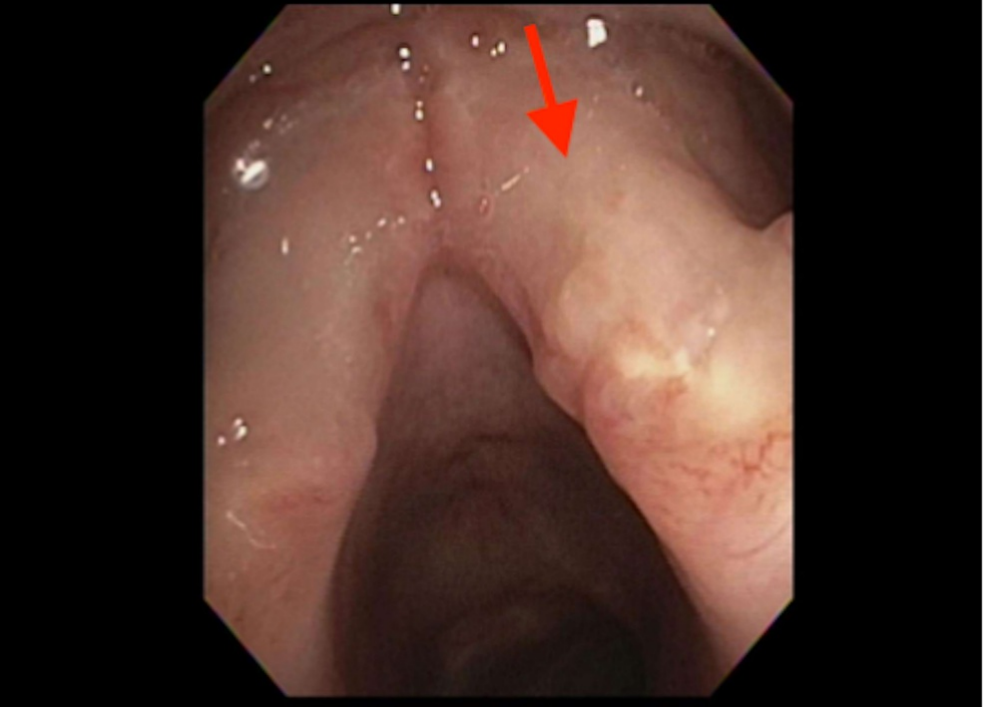
Laryngoscopy showing vocal cords with an infiltrative nodular process.

Vocal cord evaluation showed bilateral infiltrative nodular process (Figure 2). A biopsy was performed and showed acute and chronic inflammatory changes with no evidence of lymphoma. The outside biopsies were also reviewed at our institution and did not support the diagnosis of lymphoma.

**Figure 3:**
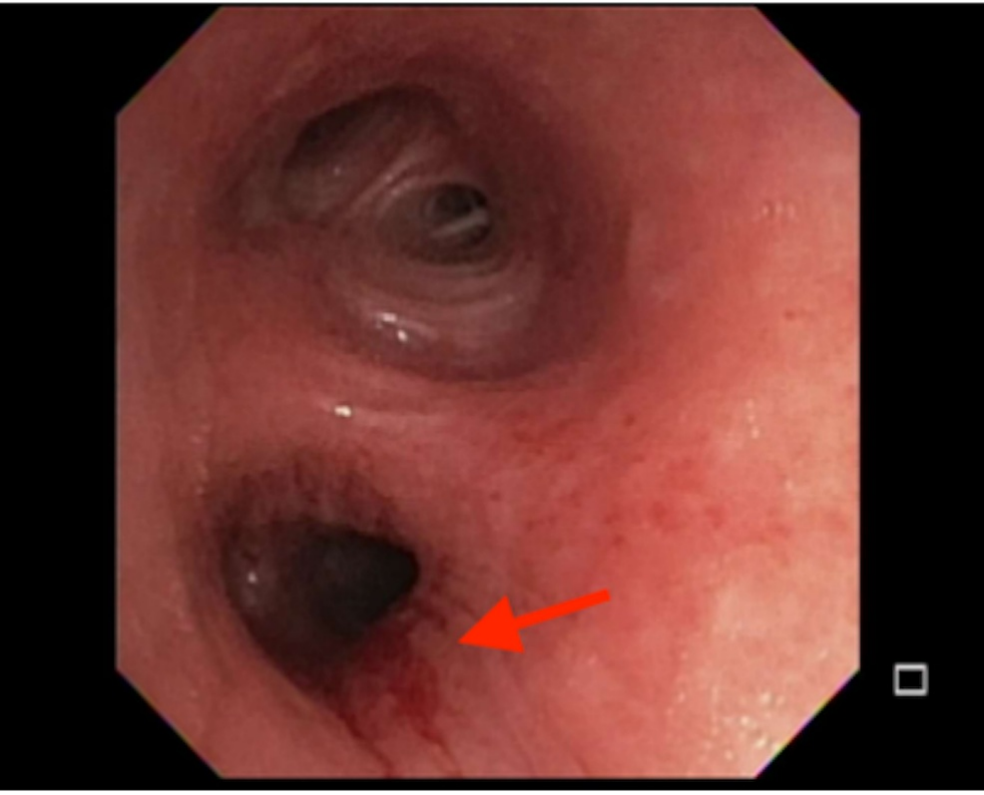
Bronchoscopy showing submucosal nodular infiltration of the left main and lower lobe bronchus.

The patient underwent bronchoscopy which demonstrated similar submucosal nodular infiltration of the left main and lower lobe bronchus (Figure 3). Endobronchial biopsy of these areas revealed granulomatous inflammation, and the underlying stroma showed diffuse infiltration by lymphocytes, plasma cells, occasional polymorphs along with epithelioid granulomas, Langhans giant cells and caseous necrosis. Histopathologic findings and acid-fast bacilli culture confirmed Mycobacterium TB and a diagnosis of laryngeal TB was made. A standard six-month treatment with a combination of isoniazid, rifampicin, pyrazinamide, and ethambutol was started and showed resolution of the symptoms and improvement of the mass.

## Discussion

Laryngeal TB results from pulmonary TB, although it might be localized in the larynx as a primary lesion without pulmonary involvement. Primary laryngeal TB without pulmonary disease is presumed to arise from direct invasion of the larynx via inhalation. It is very rare and accounts for less than 1% of all cases of tuberculosis [2,3]. The main presenting symptom of laryngeal TB is hoarseness that may be accompanied by dysphagia, odynophagia, cough, stridor, or nonspecific symptoms such as weight loss, fever, and fatigue. On laryngoscopy, visual appearance is variable and lesions may appear as ulcerofungative, ulcerative, nonspecific inflammations, or polypoid masses [4,5]. These lesions are seen most frequently in the posterior glottis and anterior glottis, but have been seen throughout the larynx. Depending on the presentation, it may resemble acute viral laryngitis or carcinoma of the larynx. Laryngeal TB is easily misdiagnosed as primary malignancy given its mass-like and infiltrative appearance as well as its relatively nonspecific presentation. Besides squamous cell carcinoma, differential diagnoses include chondrosarcoma, atypical carcinoid tumor, extranodal natural killer/T cell lymphoma, diffuse large B-cell lymphoma, paraganglioma, and adenocarcinoma metastasis. However, there are a limited number of reported cases in the literature of non-squamous carcinomas of the larynx. Primary laryngeal non-Hodgkin lymphoma is a rare condition and accounts for <1% of laryngeal tumors [6]. Besides malignancy, other granulomatous processes that can present with laryngeal involvement and that should be included in the differential include granulomatosis with polyangiitis, sarcoidosis, syphilis, and fungal infections such as histoplasmosis, blastomycosis, and coccidioidomycosis [7]. The diagnosis is confirmed with the identification of granulomatous inflammation, caseating granulomas, and acid-fast bacilli on histopathologic examination of biopsied laryngeal tissue [3]. Since laryngeal TB usually occurs in association with pulmonary TB, a chest radiograph may also show abnormalities and a purified protein derivative (PPD) skin test can be positive. Sputum microscopy is also positive in up to 20% of patients. PET-CT has the potential to identify the extent of extrapulmonary disease due to avid uptake of F-fluorodeoxyglucose by the tuberculous lesions and can be useful in the diagnosis of laryngeal TB. The primary treatment is with a regimen of multiple anti-tuberculous medications, including isoniazid, rifampin, and ethambutol, which result in a quick clinical response. Surgery is reserved for those cases with airway compromise [8].

## Conclusions

This case is a reminder that hoarseness and a growth-like lesion in the upper respiratory tract could be tuberculosis in origin and, therefore, laryngeal TB should be considered in the differential diagnosis, especially in patients from endemic regions. A full evaluation should be made to locate an active or inactive lesion elsewhere in the body.
